# Unravelling the Link between the Gut Microbiome and Autoimmune Kidney Diseases: A Potential New Therapeutic Approach

**DOI:** 10.3390/ijms25094817

**Published:** 2024-04-28

**Authors:** Diana Shu Yee Tan, Yibeltal Akelew, Matthew Snelson, Jenny Nguyen, Kim Maree O’Sullivan

**Affiliations:** 1 Department of Medicine, Centre for Inflammatory Diseases, Monash University, Clayton, VIC 3168, Australia; diana.tan@monash.edu (D.S.Y.T.); yibeltal.alemu@monash.edu (Y.A.); 2 School of Biological Science, Monash University, Clayton, VIC 3168, Australia; matthew.snelson@monash.edu; 3 The Alfred Centre, School of Translational Medicine, Monash University, Melbourne, VIC 3004, Australia

**Keywords:** ANCA, kidney disease, SCFA, glomerulonephritis, vasculitis, gut microbiota, diet therapy, autoimmunity, immune responses

## Abstract

The gut microbiota and short chain fatty acids (SCFA) have been associated with immune regulation and autoimmune diseases. Autoimmune kidney diseases arise from a loss of tolerance to antigens, often with unclear triggers. In this review, we explore the role of the gut microbiome and how disease, diet, and therapy can alter the gut microbiota consortium. Perturbations in the gut microbiota may systemically induce the translocation of microbiota-derived inflammatory molecules such as liposaccharide (LPS) and other toxins by penetrating the gut epithelial barrier. Once in the blood stream, these pro-inflammatory mediators activate immune cells, which release pro-inflammatory molecules, many of which are antigens in autoimmune diseases. The ratio of gut bacteria Bacteroidetes/Firmicutes is associated with worse outcomes in multiple autoimmune kidney diseases including lupus nephritis, MPO-ANCA vasculitis, and Goodpasture’s syndrome. Therapies that enhance SCFA-producing bacteria in the gut have powerful therapeutic potential. Dietary fiber is fermented by gut bacteria which in turn release SCFAs that protect the gut barrier, as well as modulating immune responses towards a tolerogenic anti-inflammatory state. Herein, we describe where the current field of research is and the strategies to harness the gut microbiome as potential therapy.

## 1. Introduction

There are several different types of inflammatory kidney diseases which are characterized by the development of glomerulonephritis, whereby the glomeruli of the kidneys become inflamed and are no longer capable of filtering out metabolic waste. If left untreated, these diseases can reach end-stage renal disease where life-long dialysis or kidney transplant is required for survival. Although characterized as glomerulonephritis, each kidney disease can have very different etiologies and treatment strategies, such as lupus nephritis, anti-neutrophil cytoplasmic antibody (ANCA)-vasculitis, IgA nephropathy, acute kidney injury, Goodpasture’s syndrome, and focal segmental glomerulonephritis.

There is increasing evidence on the importance of gut microbiota in disease. Microbial perturbations have been implicated as a risk factor in several inflammatory diseases, such as type 1 diabetes, lupus nephritis, and rheumatoid arthritis [1,2,3]. The gut microbiota, which is the community of microorganisms that reside in the human gut, plays a key role in the metabolism and fermentation of dietary fiber, including resistant starch (RS) [4,5,6]. RS is known to promote the growth and diversity of beneficial gut bacteria, which can have important effects on gut health and immune function. Prebiotics and probiotics are two dietary components that have regulatory effects on gut microbiota and overall health. Diets high in non-digestible fiber including vegetables, legumes, fruits, and whole grains are sources of prebiotics that selectively promote the growth of beneficial gut microoganisms [7]. The understanding of the relationship between SCFA-producing gut bacteria and their contributions to ameliorating autoimmune/inflammatory kidney disease is limited. This review will evaluate the relationships between gut microbiota and autoimmune kidney diseases and explores the potential for new therapies that target the gut microbiota to improve outcomes of patients with autoimmune and inflammatory kidney diseases.

## 2. Gut Microbiota in Health and Disease

**Figure 1:**
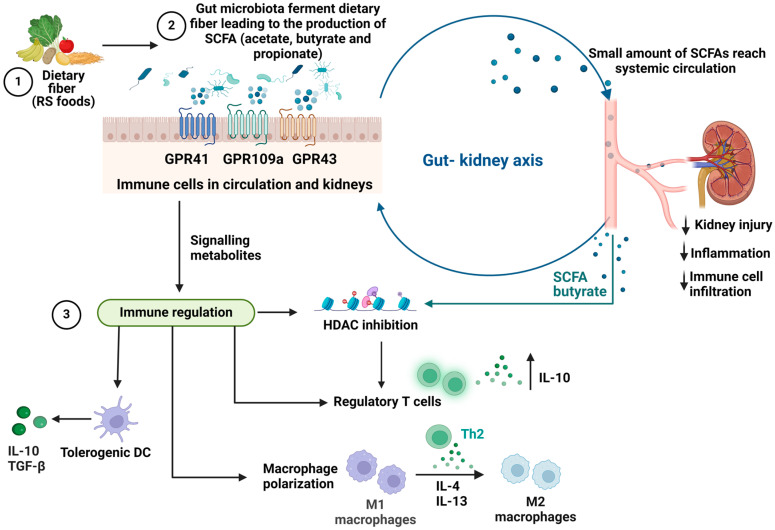
The mechanism of the effects of SCFAs on the gut–kidney axis and immune cells. (1) Dietary fiber is made up of insoluble and soluble components. (2) The uptake of dietary fiber by intestinal microbiota increases the concentration of SCFAs in the intestine and encourages the growth of SCFA-producing bacteria. Acetate, butyrate, and propionate are the most abundant SCFAs produced by gut bacteria in the large intestine, with a molar ratio of 60:20:20 respectively [13]. (3) Renal cells, immune cells, and intestinal epithelial cells have SCFA-sensing GPCR, and upon binding can alter metabolic pathways (tricarboxylic acid cycle, β-oxidation, oxidative phosphorylation), epigenetic changes through histone deacetylase (HDAC) inhibition, phenotypes (M0 to M2 macrophages), and expression of inflammatory cytokines. SCFA butyrate is also known to act directly as an HDAC inhibitor, resulting in increased histone acetylation, influencing gene expression and cellular function, hence contributing to anti-inflammatory properties [14]. SCFAs may indirectly induce renoprotection through their immunomodulatory properties such as reducing leukocyte recruitment, inflammation, and kidney injury. This figure was created with Biorender.

The gut microbiota is the community of microorganisms that inhabit the human gut. This includes bacteria, viruses, fungi, and other microorganisms, which collectively have a significant impact on human health. A key role of the gut microbiota is to metabolize and harvest nutrients from indigestible starches as they pass through the upper gastrointestinal tract (GI), which humans cannot enzymatically digest on their own [4,8] (Figure 1). RS is a type of dietary fiber that is resistant to digestion in the small intestine and reaches the large intestine mostly intact. In the large intestine, it can be fermented by gut microorganisms to produce SCFAs acting as a prebiotic to promote growth of certain beneficial bacteria such as *Bifidobacteria* and *Lactobacilli*, which thrive on the benefits of SCFA production [4,5,6] (Figure 1). RS has been shown to have several health benefits, including improved glycaemic control, increased satiety, and reduced inflammation, which may be attributed in part to the effects of SCFAs produced by gut bacteria [9,10]. Recent studies have shown that gut microbiota imbalance plays a crucial role in the development and progression of kidney diseases like lupus nephritis, diabetic nephropathy, chronic kidney disorder, and Kawasaki disease [2,5,11,12].

Dietary intervention can change the composition of the human gut microbiota [15]. A clinical trial comparing animal and plant-based diets confirmed that the human gut microbiota could be altered in a relatively brief period [16]. Animal-based diets expanded bile-tolerant microorganisms (Alistipes, *Bilophila*, and *Bacteroides*), and decreased SCFA-producing bacteria (*Roseburia*, *Eubacterium rectale*, *Ruminococcus bromii*) [4,16,17]. A comparative study examining feces of children from a rural African village in Burkina Faso found that the children had a higher abundance of phylum Bacteriodetes (also known as Bacteroidota) and depleted Firmicutes from their high fiber diets, compared with European children who consumed a typical western diet (high in fat/salt and animal products) [18]. The gut microbiota is diverse and consists of many bacterial phyla such as Bacteroidetes, Firmicutes, Actinobacteria, Proteobacteria, Fusobacteria, and Verrucomicrobia, with Bacteroidetes and Firmicutes being the two most dominant phyla, representing over 90% of the gut microbiota [19,20,21]. The balance and diversity of these microbial populations are believed to contribute to intestinal homeostasis and overall gut health. Hence, the ratio of Bacteroidetes/Firmicutes has been proposed as a marker for gut dysbiosis, with many studies reporting that patients with active disease often have gut microbiota which are more dominated by the Firmicutes phylum [8,22]. The ratio is reported to be elevated in obesity [23] and inflammatory bowel disease [24] suggesting a potential link to gut dysbiosis. In type 2 diabetes, the ratio varies and is linked to different clinical parameters such as diet, lifestyle, genetics, and environmental factors [25,26]. Alterations of the gut microbiota brought upon by ‘western diets’ have been postulated to exacerbate inflammatory diseases [4]. Consumption of a diet high in saturated fat, often found in western diets, contributes to the expansion of certain bacteria such as *Bilophila wadsworthia*, a pathobiont. Pathobionts are resident microorganisms within the host that are associated with chronic inflammatory conditions [27,28].

Increasing evidence of the role of gut microbiota in the exacerbation of autoimmune diseases have been widely reported in studies such as rheumatoid arthritis, systemic lupus erythematosus, and inflammatory bowel disease [29]. Bacterial populations with a predominantly pathobiont population, namely, Bacilli and Enterobacteriaceae [30], were found to be involved in immune dysfunction in Kawasaki disease and IBD [31,32]. Treatment regimens, disease severity, and patients’ diet and lifestyle may be contributing factors in the populations of gut microbiota observed. A more comprehensive investigation into the different factors involving patients would elucidate the influence of treatment drugs, diet, and the significance of gut microbiota in disease.

## 3. Role of SCFAs in Modulating the Immune Response

SCFAs are by-products of gut microbial fermentation through metabolization of dietary fiber and glycosylated mucins [33]. The most abundantly produced SCFAs are acetate, butyrate, and propionate, which have been shown to interact with G protein-coupled receptors (GPCRs) in the gut and other tissues [33,34]. GPCRs are a class of cell surface receptors that are involved in many important physiological processes, including the regulation of the immune system. In the gut, SCFAs have been shown to interact with several GPCRs, including GPR41, GPR43, and GPR109a, which are highly expressed on immune cells, including neutrophils, monocytes, and macrophages [5,34,35,36,37] (Figure 1). Activation of these receptors by SCFAs has been shown to have several beneficial effects on immune function, including the modulation of cytokine production, the promotion of regulatory T cells (Tregs), and the suppression of inflammation [33,34,35]. Following fermentation of dietary fiber, some SCFAs are able to interact with monocarboxylate transporters (MCTs) to be transported into and out of cells into the systemic circulation for use by other tissues [36].

SCFAs modulate the immune system by elucidating their effects on SCFA-sensing GPCRs on immune cell surfaces [38]. Butyrate was shown to induce extrathymic differentiation of Tregs and colonic FOXp3 Tregs through GPR43, as well as decrease pro-inflammatory cytokine gene expressions of IL-12, IL-6, and NFκB protein RelB in dendritic cells (DCs), allowing DCs to facilitate Treg differentiation [38]. Butyrate could also reprogram macrophage polarization to an anti-inflammatory phenotype by activating metabolic pathways favoring the M2 phenotype [39]. Disturbances to butyrate-producing gut bacteria by broad-spectrum antibiotics could perturb intestinal macrophages, shifting them to a pro-inflammatory state, highlighting how SCFA-dependent pathways are required for immune homeostasis [39].

## 4. Gut–Kidney Axis

Kidney disease is associated with increased intestinal permeability where endotoxin (a component of the gut bacterial cell wall) translocates across the intestinal barrier into the blood stream, activating immune responses. Although data on the gut–kidney axis are sparse, evidence shows kidney injury leads to the accumulation of urea and other uremic toxins which can translocate into the intestinal lumen and perturbate the commensal bacteria [40]. Urea is converted to ammonia, which damages tight junctions in the intestine, contributing to intestinal permeability. Conversely, the gut microbiota is also a source of metabolites that enhance the intestinal barrier, most notably anti-inflammatory SCFAs such as butyrate, propionate, and acetate. A diet high in non-digestible carbohydrates, such as RS, promotes the growth of SCFA-producing bacteria, which are notably depleted in end-stage renal disease (ESRD), lupus nephritis, and IgA nephropathy. There is a large body of evidence demonstrating that there is a significant difference in gut microbial diversity and metabolic pathways between healthy individuals and patients with inflammatory diseases. Given the role of SCFAs in maintaining immunological homeostasis both locally at the gut and systemically, it is critical to investigate the role of the gut–kidney axis in kidney diseases and test potential gut-targeted therapeutic interventions.

## 5. Role of Microbiota in Specific Inflammatory Kidney Diseases

### 5.1. ANCA-Associated Vasculitis

ANCA-associated vasculitis (AAV) is an autoimmune disease that affects small blood vessels, including those in the kidneys. AAV can be categorized into three clinical manifestations: microscopic polyangiitis (MPA), granulomatosis with polyangiitis (GPA), and eosinophilic granulomatosis with polyangiitis (EGPA), depending on the distribution of vascular inflammation, autoantigen specificity, and presence/absence of granulomatosis with asthma. AAV can be identified through clinical pathological phenotypes, such as MPA when there is an absence of granulomatosis with asthma, GPA when there is granulomatosis but absence of asthma, and EGPA when there is presence of granulomatosis with asthma [41]. Although target organs involved in AAV tend to be kidneys, skin, peripheral nerves, and the upper and lower respiratory tract, AAV can also be organ-specific depending on the AAV subtype [41]. Common clinical features between the three clinical subtypes are the loss of tolerance to autoantigen specificity [41] and the generation of autoantibodies to cytoplasmic granules in neutrophils, which are myeloperoxidase (MPO) and proteinase 3 (PR3) that are present in the circulation of most patients [42,43]. These autoantibodies are known as ANCAs [42,44]. ANCAs are thought to cause glomerular and vascular inflammation in AAV. The incidence of MPO-ANCA is predominantly found in the Asia–Pacific region, particularly the Chinese and Japanese populations, whereas GPA-associated PR3-ANCA incidence decreases towards the equator, with higher incidence in Northern Europe, India, and the Middle East [45,46].

MPO-ANCA-associated glomerulonephritis (GN) is a rapidly progressive disease with a high mortality of 80% between 5 to 12 months if left untreated [47,48,49,50,51]. The disease often results in rapidly progressive crescentic GN which leads to renal failure and eventual death. Treatment in active disease often includes a combination of immunosuppression therapies with high-dose glucocorticoids and cyclophosphamide [50,51]. Current treatment regimens have greatly reduced the significantly high mortality rate by more than 90% [50]. However, because of the toxicity of the immunosuppressant, the rate of drug-induced complications is high [52,53]. Furthermore, GN may relapse following withdrawal of treatment with corticosteroids and cyclophosphamide and often requires ongoing maintenance therapies [51,52,53]. Other therapies utilizing drugs such as rituximab directed against B cells and plasma exchange have shown promisingly higher recovery rates in clinical trials [53,54,55,56]. Novel and less invasive alternative therapies aimed at reducing inflammation in AAV or prolonging the period of remission will be a more focused and strategic approach at targeting MPO-ANCA vasculitis. Recently, the association between gut microbiota and AAV was reported in a study conducted with a Chinese cohort [30]. Differences in the gut microbiota were observed in patients with AAV with a predominantly pathobiont population, namely, Bacilli and Enterobacteriaceae [30].

Compelling evidence from preliminary studies in our laboratory on an established murine model of MPO-AAV have shown that a high fiber diet alters the ratio of Bacteroidetes/Firmicutes. HFD led to the enrichment of Bacteroidaceae, Muribaculaceae, and Tannerellaceae, which are families of SCFA-producing bacteria that come from the phylum Bacteroidetes. Prophylactic consumption of HFD ameliorated histological renal injury, which was associated with decreased glomerular immune cell (neutrophils, macrophages, and CD4+ T cell) infiltration into the kidneys [57]. That study provides a proof of concept that in the context of MPO-AAV, an RS-diet can shift the balance of Firmicutes/Bacteroidetes. One method of overcoming the variable gut mirobiota population between mice and humans is by using a fecal microbiota transplant (FMT) technique from a human donor into specific-pathogen-free (SPF) mice, which can mimic human microbiotas (humanized gnotobiotic mice) [58]. SPF mice have decreased gut diversity within their cecum compared with wild mice, possessing enterotypes correlating with low species richness (alpha diversity) and inflammation (family Bacteroidaceae and Enterobacteriaceae) [59,60]. A study using SPF C57BL/6 mice found it possible to successfully engraft 85% of the donor’s gut microbiota upon FMT, encompassing all bacterial phyla, 11/12 bacterial classes, and 58/66 bacterial genera found in the donor’s gut microbiota [58].

### 5.2. Goodpasture’s Syndrome

Goodpasture’s syndrome is also known as type I crescentic GN with linear immunoglobulin deposition or autoimmune anti-glomerular basement membrane (anti-GBM)-associated GN. The disease accounts for around 10% of cases of crescentic GN [61]. The disease is associated with humoral autoimmune responses to the non-collagenous domain of α3 type IV collagen, which is a constituent of the GBM [62]. It is recognized by the linear deposition of the anti-GBM antibody in the GBM [62]. Antibody autoreactivity results in complement activation by the Fc receptor that can cause glomerular injury by recruitment of macrophages and neutrophils to the glomerular capillaries, causing diminished renal function observed through proteinuria and hematuria [62]. Goodpasture’s syndrome can be a serious and potentially life-threatening condition, and prompt and aggressive treatment is essential for the best possible outcome. Treatment typically involves plasma exchange and medications that suppress the immune system, such as corticosteroids and immunosuppressants [63].

There is currently no evidence linking Goodpasture’s syndrome to the gut microbiota. There has been one study in experimental anti-GBM in rats suggesting that SCFA treatment can attenuate pathological endpoints in anti-GBM disease. In particular, butyrate was able to reduce urine protein, serum creatinine, and glomerular crescent formation [64]. However, certain medications such as immunosuppressants (cyclophosphamide and prednisolone, see Section 6) used to treat Goodpasture’s syndrome can affect the gut microbiota. This can lead to an increased risk of developing infections and other complications. Thus, alterations in gut microbiota composition and function may contribute to the development of Goodpasture’s syndrome.

### 5.3. IgA Vasculitis

IgA vasculitis, also known as Henoch–Schonlein purpura, is a type of vasculitis that primarily affects small blood vessels, including those in the skin, kidneys, and gastrointestinal tract. It is caused by the deposition of IgA immune complexes in the blood vessel walls, leading to inflammation and damage to the blood vessels. The exact cause of this condition is unknown, but it is thought to be related to an abnormal immune response triggered by an infection or other environmental factors. Symptoms of IgA vasculitis can include a rash on the skin, joint pain and swelling, abdominal pain, and blood in the urine. Diagnosis is typically based on a combination of clinical symptoms and laboratory tests, including blood tests and a kidney biopsy. Neutrophils are the predominant cells found in cutaneous and gastrointestinal biopsies of patients with IgA vasculitis [65]. Studies have shown that NETs play a role in the pathogenesis of IgA vasculitis [66]. In particular, NETs have been found to be involved in the formation of immune complexes and in the activation of the complement system, which can lead to tissue damage [67,68]. It is possible that NETs are activated by bacterial products that escape through tight junction proteins between the gut epithelial cells. Both LPS from Gram-negative bacteria and CpG can activate NETs through the pattern recognition receptors TLR4 and TLR9, respectively [69,70,71,72]. Treatment for IgA vasculitis may include medications to reduce inflammation and control symptoms, such as non-steroidal anti-inflammatory drugs (NSAIDs), corticosteroids, and immunosuppressive drugs, causing further perturbations of gut bacteria. In most cases, the condition resolves on its own within a few weeks to months, but some individuals may experience long-term kidney damage or other complications.

The relative abundance of Actinobacteria in patients with IgA vasculitis is lower compared with healthy controls [73]. As IgA vasculitis can be heterogenous in presentation, that study categorized patients according to organ types affected. Interestingly species diversity of gut bacteria changed according to the different categories. Those patients that mainly had skin and manifestations with no gut involvement had a dominance of the Actinobacteria family, while overall diversity was decreased in all patients with IgA vasculitis compared with healthy subjects. A study of inherited microbiota from mother to children in IgA vasculitis demonstrated that the children’s gut microbiome was strongly associated with the mothers’ [74]. In particular, the abundance of both *Magamonas* and *Lacotbacillus* was positively correlated with the mother’s abundance of the same gut bacteria. A positive correlation between clinical factors and the bacterial species from children with IgA vasculitis was found. *Alisitipes putredinis* was associated with fibrinogen, and *Bifidobacetrium longum* was associated with partial thromboplastin time (PPT, an indication of how long it takes blood to clot). *Dialister* abundance was decreased in children with IgA vasculitis. This finding agrees with other studies that have shown that *Dialister* abundance is also decreased in other inflammatory diseases such as eczema in children. A reduction in the abundance of *Ruminococcus* was also noted in children with IgA vasculitis. This is an important finding as *Ruminoccous* is responsible for the production of the SCFAs butyrate and propionate, which are anti-inflammatory in terms of T-cell activation. In agreement with these findings, another study in children found *Ruminococcus* was the most abundant bacteria in convalescent children whereas *Veillonella* was associated with acute IgA vasculitis [75]. This suggests that *Ruminoccous* is associated with gut health, and methods to enrich for *Ruminoccous* either through diet or prebiotics may be beneficial in IgA vasculitis.

### 5.4. IgA Nephropathy

IgA nephropathy, also known as Berger’s disease, is a kidney disease characterized by the deposition of pathogenic immunoglobulin A1 immune complexes in the glomeruli of the kidney. The disease involves mesangial proliferation and the deposition of inflammatory responses by the infiltration of macrophages, monocytes, and T cells, leading to damage and scarring of the kidney tissue. The exact cause of IgA nephropathy is not fully understood, but it is believed to be related to bacterial infection and autoantibodies specific for IgA1 that cause the formation of IgA1 immune complexes in the circulation, some of which deposit in the kidneys, resulting in glomerular injury [76,77]. Recent research has also suggested a possible link between IgA nephropathy and the gut microbiota.

Several studies have found alterations in the gut microbiome of individuals with IgA nephropathy, including a decrease in beneficial bacteria and an increase in pathogenic bacteria. Imbalance of gut microbiota populations is often observed between populations of Bacteroidetes and Firmicutes when compared between IgA nephropathy patients and healthy controls [78,79,80]. The gut mucosal system serves as a barrier between the external environment and the body’s internal tissues. IgA is predominantly found in mucosal secretions, including those in the gastrointestinal tract. Thus, IgA is crucial in the regulation of immune defence. The dysfunction of intestinal barrier can lead to increased intestinal permeability, which may allow the translocation of bacterial products and antigens into the bloodstream, triggering an immune response and influx of IgA1 deposition in the kidney.

Overall, the link between the gut microbiome and IgA nephropathy is an area of active research, and further studies are needed to fully understand the mechanisms involved and to identify potential therapeutic targets.

### 5.5. Lupus Nephritis

Lupus nephritis is one of the most common and serious complications of systemic lupus erythematosus (SLE). It is estimated that around 30% to 60% of individuals with SLE develop lupus nephritis, and it is one of the leading causes of morbidity and death among patients with SLE [81]. The relationship between lupus nephritis and the gut microbiome is an area of ongoing research, and the exact mechanisms and interactions are not yet fully understood. While there is limited research specifically examining the Bacteroidetes/Firmicutes ratio in the context of lupus nephritis, some studies have explored the gut microbiota composition in SLE. Thus, the alteration of the gut microbiota Firmicutes/Bacteroidetes ratio is commonly used as a marker for pathological symptoms and severity. Increased intestinal permeability is normally observed in individuals with SLE, which is often correlated with changes in the gut microbiota, including Firmicutes/Bacteroidetes ratio. However, the Firmicutes/Bacteroidetes ratios in SLE and lupus nephritis are not consistent across all studies [82]. Additionally, alterations in the gut microbiota composition, including changes in the Bacteroidetes/Firmicutes ratio, may be influenced by various factors such as diet, medication, disease activity, and individual variations. Therefore, it is challenging to draw definitive conclusions about the role of the Bacteroidetes/Firmicutes ratio specifically in SLE. A dominance of *Ruminococcus* in patients with SLE is associated with an increased number of circulating Tregs;and the abundance of *Ruminococcus gnavus* was reported to be five times greater than that seen in healthy controls [83,84]. SLE patients with active disease according to the Systemic Lupus Erythematosus Disease Activity Index (SLEDAI) have an increased titer of anti-*Ruminococcus gnavus* antibodies in their serum. Studies using mice models of lupus have demonstrated that probiotics containing both *Lactobacillus rhamnosus* and *Lactobacillus delbruekii* are able to downregulate microRNAs (miRNAs) associated with biomarkers in SLE. miR-181a, which can modulate T-cell receptor signaling and control B- and T-cell differentiation is increased in the serum of patients with active SLE [85]. Peripheral blood mononuclear cells from patients with SLE incubated with both *Lactobacillus rhamnosus* and *Lactobacillus delbruekii* had significant downregulation of miR-181a and miR-155, another miRNA associated with disease in SLE patients. This suggests that prebiotics containing both *Lactobacillus rhamnosus* and *Lactobacillus delbruekii* may have the potential to reduce inflammation in SLE.

### 5.6. Membranous Nephropathy

Membranous nephropathy (MN), also known as membranous GN, is an autoimmune kidney disease characterized by inflammation and damage caused by immune deposits in the glomerular filtration barrier leading to proteinuria and renal failure [86]. One of the primary features of MN is the deposition of immune complexes. Circulating autoantibodies of the IgG4 subclass target their respective antigens such as phospholipase A1 receptor 1 (PLA2R1) and thrombospondin type 1 domain-containing protein 7A (THSD7A), leading to the formation of immune complexes that deposit in the GBM and trigger an inflammatory response and subsequent renal injury [86,87]. The relationship between gut microbiota and its role in MN is an area of emerging research. A comparative study between MN patients and healthy controls reported distinct gut microbial signatures [88], suggesting a role of gut microbiota in the pathogenesis of MN. While findings are not consistent across all studies, MN patients exhibit some similarities in the alterations in the diversity and richness of specific bacterial phylum compared with healthy controls. At the phylum level, there is a decreased abundance of Bacteroidota and increased abundance of potentially pathogenic bacteria Pseudomonadota and Actinobacteria that are often associated with gut perturbations [88,89,90]. Further investigations on the abundance of the gut microbiota composition at the genus level found similarities with a significant increase in *Escherichia–Shigella* and unclassified Enterobacteriaceae, whereas there was a decrease in *Bacteroides* and unclassified Lachnospiraceae in the microbiome profiles of MN patients compared with healthy controls [88,89,90]. These studies collectively suggest a potential link between gut microbiota and MN, although further research is required to fully determine the mechanisms involved and the potential therapeutic implications. It would be beneficial to investigate whether SCFA-producing bacteria ameliorate MN, as this could be a dietary therapeutic strategy in managing the disease, or whether FMT therapy could be a novel strategy in disease protection, as there was a case study on a patient with MN reported to be in remission following FMT from a healthy donor [91].

## 6. Gut Microbiota and Interactions with Standard of Care Immunosuppressants

The critical role of gut microbiota in maintaining bodily functions is well established. How the microbiota interacts with therapeutics commonly used in standard of care for inflammatory diseases (cyclophosphamide, prednisolone, rituximab, and cyclosporine) is not well-defined. Emerging evidence suggests that microbiota and therapeutic treatments interact with each other. In particular, the gut microbiota can influence drug pharmokinetics through alteration of metabolic processing, and either increase or decrease bioavailability of drugs. An example of this is cyclosporine A, a calcineurin inhibitor which is commonly used in autoimmune diseases and in transplants to prevent rejection [92].

In the case of cyclophosphamide (CYC), it has been shown that it can alter the composition of the microbiota of the small intestine and aids in the translocation of Gram-positive bacteria into secondary lymphoid organs. It is thought that CYC injures the gut mucosal layer, damaging tight junctions and allowing the escape of bacteria [93]. Rituximab (RTX), the B-cell-depleting antibody, induces mucosal damage in a similar manner to CYC. This resulted in increased inflammatory cells and gut permeability in RTX-treated mice. In particular, *Lactobacillus reuteri* (*L. reuteri*) is reduced in the gut. In vitro studies demonstrated that *L. reuteri* inhibited inflammation in LPS-stimulated cultured mesenteric lymph node cells.

Inflammation in mice can be reduced via reintroduction of *L. reuteri* into RTX-treated mice. Prednisolone, commonly prescribed for inflammation in autoimmune kidney diseases [94], alters the gut microbiota, enriching *Anaerobacterium* species in mice studies. This was accompanied by decreases in immunomodulatory SCFAs propionate and isobutyrate. Given that patients are often on long-term doses of prednisolone, probiotics or diets that can encourage the growth of the lost bacteria are worth pursuing.

## 7. The Role of SCFAs and Innate Cells

SCFAs play a significant role in modulating the function of innate immune cells. Innate immune cells are a crucial component of the immune system that provides immediate defense against pathogens. SCFAs, which are produced through the fermentation of dietary fiber by gut microbiota, have been shown to influence the function of various innate immune cells by regulating various protein molecules such as NLRPR3 inflammasome and toll-like receptors (TLRs) within the context of the innate immune system. The inflammasome is a multiprotein complex involved in the activation of inflammatory responses. Its regulation by SCFAs, particularly butyrate, has been shown to inhibit the NLRP3 inflammasome, thus contributing to the control of inflammation [95,96]. Additionally, SCFAs act as energy substances to preserve the intestinal barrier and homeostasis and inhibit autophagy, and as histone deacetylase inhibitors to suppress the NLRP3 inflammasome [96]. SCFAs also modulate the activity of TLRs, a family of proteins that play a crucial role in recognizing microbial components, hence regulating the innate immune response to pathogens.

The influence of SCFAs on TLR signaling has been demonstrated in studies where TLR3 and TLR4 activation were downregulated, particularly by butyrate and propionate [97,98]. Butyrate and propionate have been shown to reduce the production of proinflammatory cytokines such as tumor necrosis factor alpha, interleukin 6, and interleukin 1 beta, which are mediated through the inhibition of nuclear factor kappaB (NF-κB) induced by TLR activation [98,99]. Additionally, butyrate has been found to modulate the expression of TLR4 and the phosphorylation of mitogen-activated protein kinases and NF-κB in colon cancer cells [97]. These findings suggest that butyrate and propionate are involved in the modulation of immune responses and inflammation through the regulation of TLR3 and TLR4 activation. Microbial triggers or dysregulation of the immune response mediated by TLR ligation can contribute to the development and exacerbation of kidney disease. TLR4 is well known for its role in recognizing LPS, a component of the Gram-negative bacterial cell wall. Studies have shown that there is an upregulation of TLR4 in CKD [100] and activation of TLR4 by LPS triggers a pro-inflammatory response that contributes to the development and progression of kidney pathologies [101]. Activation of TLR4 and TLR9 has been demonstrated to contribute to neutrophil recruitment and subsequently the exacerbation of autoimmune ANCA-associated GN [102,103]. Furthermore, TLR2, TLR4, and TLR9 have been shown to be involved in both glomerular and tubulointerstitial compartments of the kidneys in patients with AAV, with TLR4 being the most prominent, suggestive of its central role in the inflammatory processes associated with AAV in the kidneys [104]. Understanding the roles of TLRs in kidney diseases is crucial for the development of targeted therapeutic strategies. Modulating TLR signaling via SCFA interactions may represent a potential approach to mitigating inflammation and alleviating kidney injury. However, it is important to note that the effects of SCFAs may vary depending on the cell type and context, as they have been shown to increase TNFα-induced inflammation in lung mesenchymal cells [105]. Further research is needed to fully understand the mechanisms and potential therapeutic applications of SCFAs in modulating inflammatory responses. Nonetheless, the contribution of SCFAs to the promotion of an anti-inflammatory environment, influencing downstream signaling pathways associated with TLR activation, is promising. By regulating TLR signalling, SCFAs contribute to the fine-tuning of immune responses, helping to maintain a balance between effective defence against pathogens and prevention of excessive inflammation or inappropriate immune reactions.

Neutrophils are the key mediator of injury in AAV. Autoimmunity to the major neutrophil enzyme MPO or PR3 results in the generation of ANCA, which binds to activated neutrophils and triggers a unique form of pathological cell death termed “neutrophil extracellular traps” (NETs) or NETosis. NETs release webs of DNA containing injurious enzymes that cause inflammation of the blood vessels depositing the autoantigen MPO or PR3. The relationship between neutrophils and the gut microbiota in the generation of autoimmunity in AAV remain to be explored. Neutrophils are highly influenced by microbial metabolites, particularly butyrate, which have been found to inhibit NETosis [106]. It is well established that administration of SCFAs or promoting the growth of SCFA-producing gut bacteria skews the inflammatory response towards tolerance. The gut microbiota is capable of regulating neutrophil function including controlling the magnitude of inflammatory responses, and influences neutrophil activation and recruitment [107]. Targeting neutrophil–butyrate signaling pathways that inhibit its activation highlights the potential for new therapies to improve outcomes in patients with AAV.

In the model of experimental autoimmune ANCA-induced GN, neutrophils are known to be the primary immune cells to traffic to the glomeruli and subsequently cause glomerular MPO deposition and injury in the kidneys [108]. Neutrophils are key players in the innate immune system. They are the most abundant type of leukocytes and play a crucial part in the immediate response to infections. SCFAs have been shown to inhibit neutrophil activation through various mechanisms. SCFAs, particularly propionate and butyrate, were demonstrated to downregulate the production and release of proinflammatory mediators by neutrophils such as nitric oxide and pro-inflammatory cytokines TNFα and cytokine-induced chemoattractant 2αβ [109]. This effect is mediated by the inhibition of HDAC activity and NF-κB activation [109]. HDAC plays a role in the formation of NETs, which are released by activated neutrophils [110], involving the citrullination of histones by peptidyl arginine deiminase 4 [111]. Studies have shown that butyrate-mediated inhibition of HDAC can enhance the differentiation and function of Tregs [38], thereby contributing to immune tolerance and regulation of inflammation (Figure 1). In the context of ANCA vasculitis, butyrate-mediated HDAC inhibition could affect the expression of genes involved in neutrophil activation and NET formation, thereby impacting NETosis.

Furthermore, activation of the GPR43 receptor by SCFAs has been found to induce a chemotactic response in neutrophils [112,113]. The GPR43 receptor has been shown to be expressed in renal tissues [114] and identified as a key player in the activation of neutrophils [115]. SCFAs, especially acetate and propionate, are able to activate GPR43 on the surface of neutrophils and modulate the chemotaxis of neutrophils to and from sites of infection or inflammation [113] through the expression of chemokine receptors including CXCL1 and CXCL2 on the neutrophils [113]. By modulating the production and responsiveness to chemokines, SCFAs may contribute to the regulation of kidney inflammation, potentially limiting excessive neutrophil recruitment and activation and consequently dampening inflammatory responses and disease. More recently, studies on anti-GBM GN in rats demonstrated that treatment with SCFAs, particularly butyrate, ameliorated disease severity with a decrease in T-cell activation and an increase Treg cell differentiation [64]. While mechanisms by which SCFAs dampen immune responses and GN were not investigated, it is plausible that the interaction between butyrate and GPR43 expressed on renal cells caused the increase in Treg population and played a significant role in the development of GN.

## 8. SCFAs Modulate Regulatory T Cells

SCFAs have been shown to play a significant role in the regulation of Tregs’ differentiation and function. SCFAs, particularly butyrate, have been found to promote the differentiation of naive T cells into effector T cells or Tregs, depending on the immunological milieu. This process occurs through the inhibition of HDACs, leading to increased histone acetylation and gene transcription associated with Treg differentiation and regulation of the mTOR-S6K pathway [116] (Figure 1). As Tregs play a pivotal role in regulating the immune system and maintaining immune tolerance, the involvement of SCFAs is crucial in the promotion of immune homeostasis and regulation of immune responses, particularly in ANCA-associated vasculitis, where disease is the result of autoimmunity to the autoantigen. Research has consistently shown that SCFAs, particularly butyrate, enhance the suppressive function of Tregs by increasing the production of anti-inflammatory cytokine IL-10 and transforming growth factor beta (TGF-β) [116,117,118] (Figure 1). SCFAs produced by the gut microbiota have been shown to influence the migration and homing of Tregs to sites of inflammation and tissue damage by regulating the expression of chemokine receptors on Tregs, particularly the key receptor CCR4 [119], thereby guiding their migration to specific tissues or lymphoid organs [120] (Figure 1). The dysregulation of chemokine receptors has been implicated in various autoimmune diseases, including multiple sclerosis, rheumatoid arthritis, type 1 diabetes, autoimmune thyroiditis, Graves’ disease, and Addison’s disease [121,122,123,124]. These receptors and their ligands play a critical role in the recruitment and trafficking of immune cells to affected organs, contributing to the pathogenesis of these diseases. Studies have demonstrated the importance of Tregs in the maintenance of tolerance to MPO in the experimental model of MPO-AAV [125]. However, the role of chemokine receptors on Tregs in kidney disease has not been investigated. It is possible that the dysregulation of chemokine receptors via SCFA interactions in AAV may impact Treg function, contributing to the pathogenesis of the disease.

## 9. Use of Animal Models in Microbiome Studies—Considerations for Translation into Human Studies

Animal models play a crucial role in microbiome studies, providing controlled systems to investigate the complex interactions between microbial changes and disease. The microbiota composition and function can vary significantly between animal models and humans. Thus, selecting appropriate animal models and experimental designs is imperative to closely reflect human pathophysiology. Animal models are often used to test the efficacy of potential microbiome-targeted therapies, such as dietary intervention [8,11], antibiotic treatment [126], FMT [127], and administration of microbial metabolites such as SCFAs [64]. These interventions were investigated for their ability to modulate gut microbiota and ameliorate disease severity. Animal models allow the induction of specific diseases or conditions and the study of how alterations in the microbiome may contribute to disease pathogenesis.

### 9.1. Differences in Gut Microbiota Diversity between Human and Mouse Gut Anatomy

Although the gastrointestinal tracts in both species are similar, it is important to understand the critical differences when studying the gut microbiota in mice digestive tracts. In terms of intestinal surface area versus body surface area, both species are similar. However, where the difference occurs is over the anatomical structure of the gut. For example, the length ratio of the small intestine to colon is 7 in humans compared with 2.5 in mice, with the mouse cecum being larger than the human cecum, which allows them to process extra nutrients from indigestible fiber with greater fermentation capacity [128]. The location of fermentation of fiber by gut bacteria in the mouse system is compartmentalized in the cecum in comparison to the fermentation of gut bacteria in humans which occurs in the colon [129]. The physiological consequence of this is that mice have an expanded cecum capacity allowing them to digest significantly more undigestible fibers in their diet relative to humans. The differences do not end there; in terms of the histological structure of the intestinal wall, mice have much more elongated villi than humans, hence an increased surface area allowing enhanced absorption of nutrients [129].

Given the anatomical differences between mice and humans, it is not surprising that this would provide different ecological niches for gut bacteria, favouring the growth of some families over others. Despite this, the two species share 90% similarity in phyla and 89% in genera [130]. However, it is important to be aware that a large number of bacterial families found in mice are not present in humans. Mice microbiota composition favours a greater Bacteroidetes/Firmicutes ratio and humans have a greater Firmicutes/Bacteroidetes ratio [131].

### 9.2. Use of Germ Free/Gnotobiotic Animal Models

Germ-free or gnotobiotic animal models, which are animals that are completely lacking gut microbiota or with defined microbial communities, allow the study of the impact of microbiota on various diseases. Changes in the microbiome composition can be assessed by administrating prebiotics, probiotics, antibiotics, or FMT to tease out the effects certain families of bacteria have on disease outcomes. FMT can be used to colonize germ-free or antibiotic-treated animals to explore the ability of gut microbiota to modulate disease severity, as demonstrated by FMT from patients with hypertension [132] and end-stage renal disease [133] and mice with colitis [134] to germ-free mice.

Germ-free or gnotobiotic animal models are a critical resource for the study of the intestinal microbiota. Germ-free mice, as the name suggests, have had all their gut microbiota removed. This in itself is technically challenging, requiring a dedicated facility where mice pups are removed under sterile conditions from their mothers to prevent contamination with the mother’s bacteria flora [135]. In contrast, gnotobiotic animals allow mice to be colonized with specific microbiota, allowing researchers to examine the specific characteristics and importance of specific strains of bacteria. The most common use of germ-free mice is as a recipient for FMT studies. However, these models are not without their limitations. The raising of mice in a sterile microisolator can lead to abnormalities in their development as well as an abnormal immune system [136]. Work carried out in these germ-free facilities comes at a great cost as they require dedicated staff and space for the microisolators. Given the cost, an important alternative is to use antibiotics to deplete the gut bacteria. There are different combinations used by different laboratories but the most common cocktails use varying concentrations of ampicillin, vancomycin, and gentomycin [137,138]. Some of the issues with this approach include side effects from the antibiotics which can affect metabolic processes and disturbances in immune cell functions [139].

### 9.3. Housing Consideration When Using Mouse Models for Microbiota Studies

Experimentation in animal models requires controlled experimental conditions, allowing standardized protocols and reproducibility of results. However, factors such as housing, age, sample collection site, littermate effect, bedding, and supplier effect may influence the consistency and reproducibility of experiments [140].

Each individual cage that mice are housed in creates a unique environment. This is due to close contact with other mice and coprophagy (consumption of other mouse faeces). To reduce agistment costs in animal houses, it is common practice to put four or five mice in the same cage. Studies have shown that reducing housing density to two per cage has an effect on the gut microbiota, providing more effective antibiotic treatment than to four or five mice housed together [141]. Furthermore, fluctuations in temperature not only affect gut microbiome composition but reduce reproducibility for microbiome experiments. An example of this is that mice housed at 22 degrees Celsius have an enrichment of *Lachnospiraceae* compared with mice housed at 30 degrees Celsius, which are enriched in *Prevotellaceae* [142].

### 9.4. The Requirement for Guidelines for Studying the Microbiome

The role of the gut microbiome in varying diseases has become more apparent in recent years, gaining traction as an appealing strategy for treatment of varying diseases. With this comes an increasing need to ensure there are guidelines governing this that allow reproducibility. A critical factor for consideration in this area of research is determining causation versus association. In terms of examining the role of the microbiome in renal diseases, there is an additional confounding issue in that the relationship between kidney function and the gut microbiome is reciprocal. Urea that accumulates systemically as renal disease progresses is able to translocate across the gut epithelia where it has a direct effect on the gut microbiota. Conversely, ammonia, which is released as a by-product of urea hydrolysis, is utilized as a source of nitrogen for bacteria to synthesize critical amino acids, puramines, and sugars [143]. Therefore, when conducting microbiome studies to determine causation versus association, reverse experiments should be conducted where gut bacteria from the diseased groups of interest are transplanted via FMT into germ-free mice [133].

FMT itself needs strict guidelines. Guidelines for reporting on animal fecal transplantation (GRAFT) have been published outlining the importance of having standard protocols for the collection, downstream processing, and minus 80 degrees Celsius storage of the samples before transplant. One of the critical factors in preparation of the samples for transplant is the vehicle in which it is prepared, paired with the conditions in which it is prepared. For example, if the fecal samples are not prepared under anaerobic conditions, many of the anaerobic bacterial families will have decreased viability [144].

This gives impetus to ensuring findings in mouse studies are replicated in well-designed human clinical trials. Special consideration should be given to the microbiome-targeted interventions such as high fiber diets that have been utilized in mouse studies, to ensure effective translation into human studies.

## 10. Modulation of Gut Microbiota as Potential Therapy

Dietary fiber is critical for a healthy gut. Non-digestible fibers are fermented by gut bacteria which produce SCFAs that in turn provide an energy source for colonocytes to enhance gut barrier integrity. The epithelium of the colon receives the majority of its energy from SCFAs (over 70%), of which butyrate is the most critical. A complication with the treatment of patients with CKD is that they are often prescribed low fiber diets to limit potassium intake due to the risk of hyperkalaemia [145]. Therefore, the gut microbial composition becomes altered due to the lack of fiber.

### 10.1. Dietary Intervention

**Figure 2:**
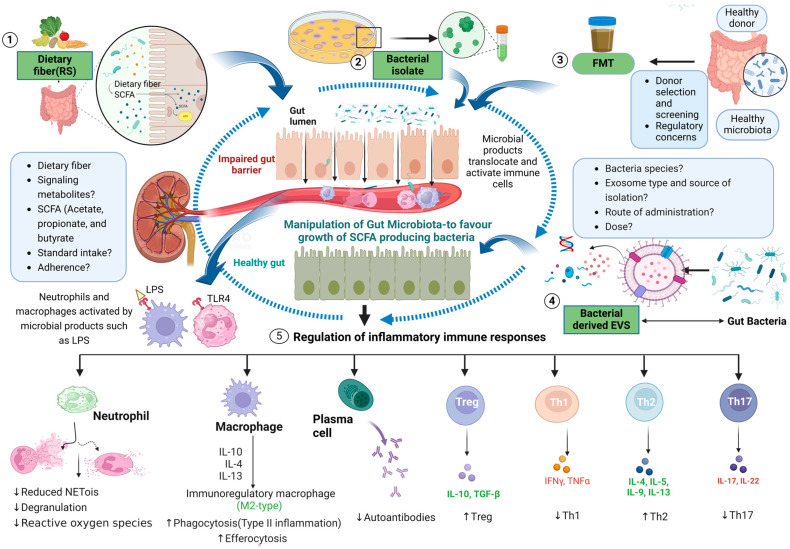
Strategies for modulating the inflammatory response during autoimmune kidney disease. These include dietary fiber intake, SCFAs (namely acetate, propionate, and butyrate), the transfer of gut microbial isolate, faecal microbial transplantation, and gut bacterial EV therapy. (1) Dietary fibers and starches that are resistant to digestion in the small intestine promote gut microbiota, and the metabolized product (SCFAs) is in turn associated with gut microbiota homeostasis. (2) The transfer of immunomodulatory microbial isolate(s) can modulate inflammation and reduce the risk of introducing pathogenic bacteria. (3) Faecal microbiota transplantation from a healthy donor also introduces gut microbiota into the patient, thereby modulating inflammation. (4) Isolated and characterized EV from gut bacteria with known immunomodulatory properties can also serve as therapeutic targets. The yellow triangle shows when the innate sensor (TLR4) on the neutrophil cell surface detects bacterial LPS triggering subsequent adaptive immune responses. (5) As a result, all these potential targeted therapies could modulate CD4+ T cell differentiation, induce the activity of Treg responses, regulate B-cell activity, and regulate neutrophils and other immune cells, all dampening the host immune responses. Type 2 responses suppress Th1 responses against the disease and skew cytokine profiles, resulting in a dominant IL-4, IL-5, IL-9, and IL-13 response produced by expanded populations of alternatively activated macrophages. Abbreviations: FMT: faecal microbiota transplantation; IFNγ: interferon-gamma; IL: interleukin; LPS: lipopolysaccharide; M2: alternative-type macrophage; NETosis: formation of neutrophil extracellular traps; SCFA: short-chain fatty acids; TLR4: toll-like receptor 4; Th: helper T cell, TNFα: tumour necrosis factor alpha. This figure was created with Biorender.

A western diet is characterized by a high intake of processed food containing fat and sucrose and low intake of dietary fibers, minerals, and vitamins, linked with several metabolic disorders and inflammatory disease [146,147]. Dietary intervention has an obvious impact on health but its composition can also modulate the microbiota profile, thereby dysregulating the host immunity. Several studies have shown that diet, microbiota, and autoimmune diseases are highly interconnected [148]. As a result, prebiotics such as dietary fiber are metabolized into SCFAs and other products which have both a direct and indirect effect on our health [149] (Figure 2).

One of the dietary recommendations is fiber, which is a non-digestible carbohydrate that bypasses degradation by the digestive enzyme and reaches the large intestine intact [150]. Resistant starch in particular is fermented slowly over time; therefore, compared with other fermentable oligosaccharides, disaccharides, monosaccharides, and polyols (FODMAPS) that contribute to unwanted side effects such as bloating and gas, RS is much more palatable as a choice. Evidence from clinical trials using high-amylose-resistant starch that has been enriched with both acetate and butyrate (HAMSAB) has shown that immune profiles in type 1 diabetes can be altered with as little as 6 weeks of supplementation. Those subjects who had the highest concentrations of SCFAs in the blood had better glycemic control [151]. Short-term studies with HAMSAB for the control of hypertension have also shown positive results with a reduction in 24 h systolic blood pressure. Importantly, HAMSAB supplementations were associated with a change in the gut microbial diversity by creating an ecological niche that favored the growth of SCFA-producing bacteria [152].

Dietary fiber intervention has a positive effect by stimulating the production of SCFA metabolites and promoting beneficial gut microbiota, leading to decreased production of uremic toxin, creatinine, and inflammatory response in people with CKD [153]. A prospective observational study involving 61 Belgian children (age < 18 years) with all stages of CKD (stage 1–5) including recipients of transplant and different etiologies (congenital anomalies of the kidneys and urinary tracts, polycystic disease, and other non-glomerular diseases) demonstrated an opposite relationship between the rise in fiber intake (g/day) and serum concentrations of different gut-derived protein-bound waste products, namely indole acetic acid (free and total), indoxyl sulfate, free p-cresyl sulfate, and p-cresyl glucuronide (free and total) [154].

Analysis of a nationwide database reported that among 14,543 participants, 25.7% of the CKD subpopulation had elevated serum c-reactive protein (CRP) (>3 mg/L) compared with the non-CKD subpopulation. Additionally, DF intake (10 g/day) (total, soluble, and insoluble DF) was significantly associated with lower serum CRP in CKD patients [155]. Another cohort of 1110 elderly Swedish men (age 70–71 years) with CKD were given a 7-day dietary fiber intake of 10 g/day higher than their usual intake. This resulted in an increase of eGFR with an average of <60 mL/min per 1.73 m and reduced mortality. Notably, participants in higher dietary fiber quartiles had lower levels of CRP (<3 mg/L) and IL-6 [156].

In a recent randomized trial, a total of 162 participants with end-stage renal disease were instructed to consume 10 g of DF (potato starch) orally each day for 8 weeks. *Lactobacillus*, *Bifidobacterium adolescentis*, and *Lactobacillaceae* were significantly increased in the DF group. Interestingly, SCFAs, namely, butyric acid, isovaleric acid, isobutyric acid, hexanoic acid, and valeric acid, were also significantly increased whilst there was no significant change for either propionic acid or acetic acid levels [157].

An observational study in Germany comprising 3193 moderately severe CKD participants utilized a food frequency questionnaire to score adherence to dietary recommendations. The study revealed that increased consumption of fiber and potassium was linked to higher eGFR and lower CRP. Conversely, higher intake of sodium, cholesterol, total protein, and sugar had the opposite effect [158]. In a follow-up study on potassium intake in a South Korean cohort, the effect of dietary potassium was assessed. Among 5064 participants with stage 2 CKD (aged ≥ 40 years), the study found that those with high potassium intake (Q3 1765.603–2364.251 and Q4 > 2364.251 mg/day) had a lower risk of CKD development and a decrease in eGFR (<60 mL/min/1.73 m^2^) [159].

RS has been proposed as a treatment for CKD patients to modulate the microbiota in the gut by promoting the proliferation of favourable gut bacteria and increasing the production of metabolites including SCFAs [5]. It can be degraded by a diverse group of microbes such as primary degraders (*Ruminococcus bromii* and *Bifidobacterium adolescentis*) and secondary degraders (*Eubacterium rectale*, *Roseburia*, and *Butyrivibrio*) as well as *Bacteroides thetaiotaomicron*, which can generate metabolites like butyrate, lactate, and acetate [160].

Studies in experimental models of disease and humans with kidney diseases have shown that RS intervention increases the concentrations of SCFAs and diversity of gut microbes. For instance, rats fed a diet containing high-amylose maize starch (5% RS type 2), compared with a calorie control group, showed an increase in fecal SCFA after a 2-week intervention. Additionally, lower levels of pH and blood urea were observed [161]. Similar interventional research using RS [high-amylose maize RS (59%)] for a duration of 28 days in a mouse model of CKD (via nephrectomy) demonstrated a slow progression of CKD. The gut bacterial diversity was also higher in RS groups. However, BUN/creatinine levels were not significantly altered in CKD mice supplemented with RS compared with control mice fed a calorie-matched diet [162].

In a randomized trial, 75 patients (18–80 years) with early diabetic nephropathy were treated with 50 g of high RS for 12 weeks. This resulted in a reduction in glucose levels and an increase in serum uric acid and superoxide dismutase levels. However, the diet was unable to reduce inflammation markers IL-6 and TNFα [163].

Likewise, in a randomized control trial conducted in Iran, 20 patients undergoing haemodialysis or end-stage kidney disease were given biscuits comprising 20 and 25 g/d of high-amylose maize RS type 2, during the first and second month respectively. Interestingly, the 16s DNA sequencing from fecal samples revealed a significant increase of *Faecalibacterium bacteria* but not the genera *Parabacteroides*, *Bifidobacteria*, *Ruminococcus*, or *Prevotella* [164]. As seen in the previous studies, neither TNF-α nor IL-6 were significantly altered with this diet.

### 10.2. SCFAs as Potential Therapeutic Agents

SCFAs, namely, acetate, propionate, and butyrate, represent the main metabolites generated by the gut microbiota, all of which have immunomodulatory functions [165]. As mentioned earlier, the anaerobic fermentation of DF and RS by gut microbiota produces SCFAs. As a result, dietary fiber is strongly associated with increased SCFA production [166]. Thus, dietary intervention, SCFAs, and gut microbiota profile, function, and metabolism are highly interconnected with each other. SCFAs warrant investigation as potential therapeutics to either restore gut homeostasis via protective effects on the gut barrier or to modulate inflammatory cells (Figure 2).

SCFAs produced by gut bacteria provide protection to the gut epithelium. SCFAs are recognized by specific GPCRs in the colon. Via these receptors, butyrate, for example, is able to aid in enhancing the mucus layer by increasing production of mucin 2. Acetate produced by *Bifidobacterium*, an SCFA-producing bacteria, provides energy for both propionate and butyrate-producing bacteria, an example of a postive feedback loop whereby one bacteria releases SCFA to enhance the growth of another [167]. Healthy adult blood is estimated to have concentrations of 100–150 µmoL/L for acetate, 4–5 µmoL/L for propionate, and 1–3 µmoL/L for butyrate [168] with a molar ratio of 60% acetate, 20% butyrate, and 20% propionate in the large intestine [169]. Acetate and propionate are mainly induced by Bacteroidetes (Gram-negative), while butyrate is predominantly triggered by Firmicutes (Gram-positive) gut microbial phyla [170].

In vitro butyrate and propionate are able to promote the production of Tregs from naïve T cells in co-culture with DCs. However, Th1, Th2, and Th17 cytokines (IL-4, IL-13, IL-17, IFN-γ) remained unchanged upon butyrate treatment [38]. Butyrate has potent anti-inflammatory effects that are involved in intestinal homeostasis and energy metabolism [171,172]. SCFAs also regulate the mTOR–S6K pathway and Tregs by promoting IL-1, IFNγ, and/or IL-10 [116]. Interestingly, a reduction in anti-CD3-induced inflammation occurred in the acetate-treated group, in an IL-10-dependent manner [116].

Li et al. demonstrated that butyrate significantly inhibited the formation of NETs and neutrophil-associated inflammatory mediators including chemokines (CXCL1, CCL3, CCL4, and IL-8), CCL20, CXCL9, and MPO, which were significantly decreased in patients with IBD [106]. Nephrectomised rats receiving sodium butyrate (400 mg/kg/day) in drinking water had markedly improved insulin resistance and significantly reduced circulating LPS levels. This was accompanied by restoration of decreased IL-10 levels compared with the control groups. Markers of kidney injury were also significantly decreased, with lower levels of serum urea and proteinuria in the treatment groups; however, there was no effect on creatinine levels [173]. This suggests that treatment with SCFAs, in particular butyrate, may be of potential therapeutic benefit in kidney diseases.

A study in humans has shown that SCFAs given as apple cider vinegar or through RS inulin (to induce bacteria to release SCFAs) can modulate immune cells in healthy individuals. After 3 weeks of the intervention, B cells were lower on the high-SCFA diet compared with those on a low-SCFA diet. Likewise, Th1 cells were lower in the high-SCFA diet compared with the low-SCFA diet [174]. This provides proof of concept that SCFAs can influence different populations of immune cells and may be of potential therapeutic benefit in the treatment of inflammatory kidney diseases. Although a high fiber diet containing RS can produce large amounts of anti-inflammatory SCFAs, it is difficult for patients to maintain these diets. This is evidenced by the increasing level of obesity in the developed world. Although it is known that a change of diet can reduce weight and encourage a healthy gut, many patients are unable or unwilling to make these changes. Therefore, administration of SCFAs themselves rather than via diet may be an attractive alternative for many non-compliant patients who do not follow prescribed diets.

### 10.3. Fecal Microbiota Transplants as Therapy

FMT has become an emerging attractive therapeutic agent with the successes seen in treatment for IBD. There is now growing interest in extending these findings to target autoimmune inflammatory diseases and cancer. FMT has been demonstrated to restore altered microbiota, reconstruct the gut micro-ecosystem, and modulate both the innate and adaptive arms of the immune system, leading to therapeutic outcomes for different diseases [175]. Among these, it has been very successful and extensively used for treating *Clostridioides difficile* infection implicated in IBD [176].

The principle behind FMT as a form of therapy is to restore altered gut microbial ecology by replacing it with fecal microbiota from a healthy individual. The donor is heavily screened and can be chosen by the patient (e.g., a family member) or from a biobank. Screening includes ensuring none of the donors have infections (particularly, hepatitis A, B, and C, HIV, syphilis, helminth, or other parasite infections) or have a gastrointestinal disorder. Importantly donors are screened to make sure they have not had any antibiotic treatment which can deplete healthy gut bacteria.

There have been multiple animal studies of kidney disease that demonstrate that FMT can alleviate renal disease endpoints (kidney histology and functional injury evidenced by lower serum creatine or albuminuria/proteinuria). The gut microbiota composition affects the intestinal barrier integrity and accumulation of harmful metabolites such as urea, indoxyl sulfate (IS), and p-cresyl sulfate (PCS), leading to accumulation of immune complexes that cause damage to the renal parenchyma in renal diseases such as lupus nephritis [177]. FMT, once transferred, has been shown to rapidly normalize the microbial community structure within 24 h after administration to the recipient [178]. A preclinical trial on humanized mice demonstrated that FMT modulated IgA nephropathy phenotype and inflammation via increasing serum B cell activation factor (BAFF) and decreasing CD89 cell surface expression, both of which are associated with IgA1 mesangial deposits [179].

A recent study aimed to improve CKD patients’ renal dysfunction by administering a washed microbiota transplant WMT for 3 consecutive days. Unlike conventional FMT, that study used a wash protocol in the preparation of the WMT. This consisted of repeated saline washes, microbiota purification, and centrifuging prior to transfer via either transendoscopic or nasojejunal tube. This method resulted in significant differences in microbial diversity and an improvement in renal function evidenced by improvements in blood urea nitrogen and serum creatine. Interestingly, some gut genera, including *Eubacterium coprostanoligenes*, *Anaerostipes*, *Monoglobus*, and *Dorea*, demonstrated a significant increase while *Hungatella* genera was decreased after FMT [180].

Despite the tremendous achievements that have been made so far, optimization of protocols and the potential metabolic and immunological consequences during host–microbiota interaction has not been clearly established [178]. Additionally, a disease-specific approach may be needed for donor selection and FMT in CKD patients, including characteristics of ideal donors, dose, frequency, route of administration, etiology and stage of kidney disease, and the need for the aforementioned recommended diets [181]. This highlights the importance of conducting standardized research as outlined in the GRAFT guidelines.

### 10.4. Transfer of Immunomodulatory Microbial Isolates as Therapy

FMT has an obvious beneficial effect in several inflammatory diseases, based on the level of success treating patients with IBD. However, the administration of whole fecal product may have a risk of introducing unexpected pathogenic bacteria into the gut. To reduce such risk, researchers have been investigating an alternative approach by administering specific bacterial strains extracted from healthy stool [182]. Importantly, depending on the strain, probiotics could have distinct molecular mechanisms and effects on modulating inflammation involved in both innate and adaptive immunity [147]. As discussed earlier [180], it can also be hypothesized that certain gut microbiota may not provide benefits, as indicated by a reduction in specific genera following FMT rather than an increase in protective immunomodulatory families of bacteria.

Although there have been limited studies of specific microbial isolates in patients, there was a randomized clinical trial aimed at observing the modulation of the intestinal microbiota in healthy adults. Some gut bacteria like *Akkermansia muciniphila*, *Bifidobacterium*, and *Faecalibacterium prausnitzii* are associated with beneficial effects that can restore the impaired gut barrier in IBD [7]. For instance, consumption of the probiotic strain *Bifidobacterium bifidum* for 4 weeks isolated from fecal samples obtained from a healthy woman resulted in a significant increase in the Ruminococcaceae and Rikenellaceae bacterial families, but a decrease in the Prevotellaceae family following administration [183]. In contrast, mono-colonization with segmented filamentous bacteria in a mouse model of MS exacerbated autoimmune demyelination through the triggering of myelin-specific CD4+ T cells and autoantibody-producing B cells [184].

### 10.5. Bacterial Extracellular Vesicles

Extracellular vesicles (EVs) in health and disease have been an area of increasing interest. EVs are a heterogeneous group of lipid-membrane-bound vesicles (30- to 5000-nm) secreted by almost all cell types [185]. EVs are generally categorized into three subtypes including microvesicles, exosomes, and apoptotic bodies, based on their biogenesis [186]. The biogenesis of bacterial EVs is unique from that of eukaryotes; based on structural differences, Gram-negative bacterial EVs are released from the outer membrane and inner membrane while Gram-positive bacterial EVs arise from the cytoplasmic membrane [187]. The EVs’ cargo comprises functional proteins, nucleic acids, lipids, and other bioactive molecules, thereby modulating the behaviour of recipient cells and playing a role in different physiological and pathological processes [188]. Due to their role in immune system modulation, the applications of EVs as therapeutic targets, novel drug delivery agents, and stand-alone therapeutics are being actively explored.

Exosomes have been widely proposed as natural nanocarriers for delivering functional RNAs, proteins, and synthetic drugs that can be used therapeutically. This is attributed to their compatibility, reduced toxicity, biocompatible, minimally immunogenic, and retaining targeting capacity properties [189]. However, studies are very limited in addressing their ability to regulate the gut microbiome as a therapy in autoimmune and inflammatory kidney disease. This limited understanding could be due to the highly dynamic structure and complexity of EVs as well as the secretions by different cells and tissues under physiological and pathological conditions.

While certain studies have shown the promising therapeutic potential of renal-derived exosomes, the critical role of gut bacterial-derived exosomes and the associated molecular mechanisms have received little attention. However, it is clear that bacteria–host interactions mediated by exosomes could affect the expression level of either inflammatory or anti-inflammatory factors depending on the environment [190].

Proteomics studies have discovered that glomerular podocytes and tubular cells are the principal cells secreting exosomes in the nephron [191]. Studies have indicated that hypoxia, along with hypoxia-inducible factor 1 (HIF-1), promotes exosome production in tubule epithelial cells, thereby modulating the fate of adjacent cells and consequently the extent of kidney damage [192]. Thus, it is reasonable to speculate that tubular exosomes could communicate with the gut microbiota and the gut bacterial EV in turn, with a direct therapeutic effect during pathological conditions of chronic kidney inflammation.

Gut bacterium *Akkermansia muciniphila*-derived EVs were shown to regulate mucosal inflammation by inhibiting the production of IL-6 from colon epithelial cells in an IBD mouse model [193]. *Lactobacillus* EVs have also been reported as a promising therapy for IBD and autoimmune diseases [194]. This implies that, based on compelling evidence that gut bacteria isolates can modulate the inflammatory response, elucidating bacterial EVs in autoimmune kidney disease could be a better potential therapeutic target avoiding the unnecessary administration of other components.

## 11. Limitations and Future Directions

Understanding the interactions within this complex microbiota ecosystem and elucidating the specific roles of individual microbial species and their metabolites, including SCFAs, presents a considerable challenge. While SCFAs have been shown to have immunomodulatory effects, the exact mechanisms are complex and not fully defined. Some studies have shown that perturbations of immune homeostasis by SCFAs or the gut microbiota can contribute to the exacerbation of autoimmune diseases through the differentiation and activation of pro-inflammatory Th1 and Th17 cells [116,195,196]. The role of gut microbiota in autoimmune and inflammatory kidney diseases is an area of growing research. There are limited reports on the influence of SCFAs and their interactions in molecular pathways associated with kidney pathogenesis. Despite the differences in human and mouse gut anatomy and microbiota populations, much of the evidence linking SCFAs, gut microbiota, and kidney disease/inflammation is based on animal models. It is therefore imperative that the human gut microbiota is considered in translational studies. The population of human gut microbiota can vary significantly between individuals due to factors such as genetics, diet, age, lifestyle, and environmental exposures. The variability poses challenges in extrapolating findings across varied populations, thus restricting the relevance of research outcomes to broader contexts. Longitudinal studies tracking changes in the gut microbiota and SCFA production in diverse groups of patients at different stages of disease will be crucial in understanding the interactions between the gut microbiota and kidney diseases. Bacterial EVs have generated significant interests in recent years due to their involvement in regulating the intestinal microenvironment and mediating host–microbiota interactions [197,198]. They are key players in immune modulation and the maintenance of intestinal barrier integrity, making them a potential target for clinical applications [199]. Hence, further research using multi-omics will be beneficial to explore the complexity of the molecular mechanisms and determine the interactions of EVs with cell receptors in different kidney diseases.

## 12. Conclusions

The role of the gut microbiome in autoimmune and inflammatory kidney disease is becoming an area of growing research. Given the lack of current treatments that are both effective and without toxic side effects, exploring mechanisms in which we can control inflammation and mitigate disease progression through the manipulation of gut microbiome presents potential for novel therapeutic approaches. Nevertheless, numerous questions persist regarding the utilization of gut microbiome manipulation as therapy. Therefore, future research efforts into autoimmune/inflammatory kidney diseases should also be a focus. These should include but not be limited to investigations of underlying mechanisms, identification of therapeutic targets, comparative studies of the aforementioned potential therapeutics along with their advantages and disadvantages using mechanistic animal models, as well as translational clinical trials in humans for validation of the safety and efficacy of the intervention.
